# Supplementary material for: A Blinded Investigation: Accentuated NK Lymphocyte CD335 (NKp46) Expression Predicts Pregnancy Failures
Source: Diagnostics (Basel). 2023 May 25;13(11):1845. doi: 10.3390/diagnostics13111845 (PMC10252685; doi:10.3390/diagnostics13111845)

Supplementary Figure S1. Gating strategy used to identify NK cells and NKp46 NK cell subsets. Representative flow cytometry plots from the peripheral blood of 3 study participants (with contrast type of CD335 expression). The lymphocytes were live gated during acquisition using the side and forward scatter dot plot display (A). Furthermore, by using the negative gating strategy, CD3negative lymphocyte population was identified (B). The NK cell population was further identified as CD3−CD56+

– (%NK) and differentiated into NKp46high, NKp46dim and NKp46neg subsets(C, D, E). The NKp46high subsets was also separated on CD56++ and CD56dim. In parallel to manual gating, standard invariant gating for $NKp46^{high}$, $NKp46^{medium}$, and $NKp46^{neg}$ enumeration were conducted (F).

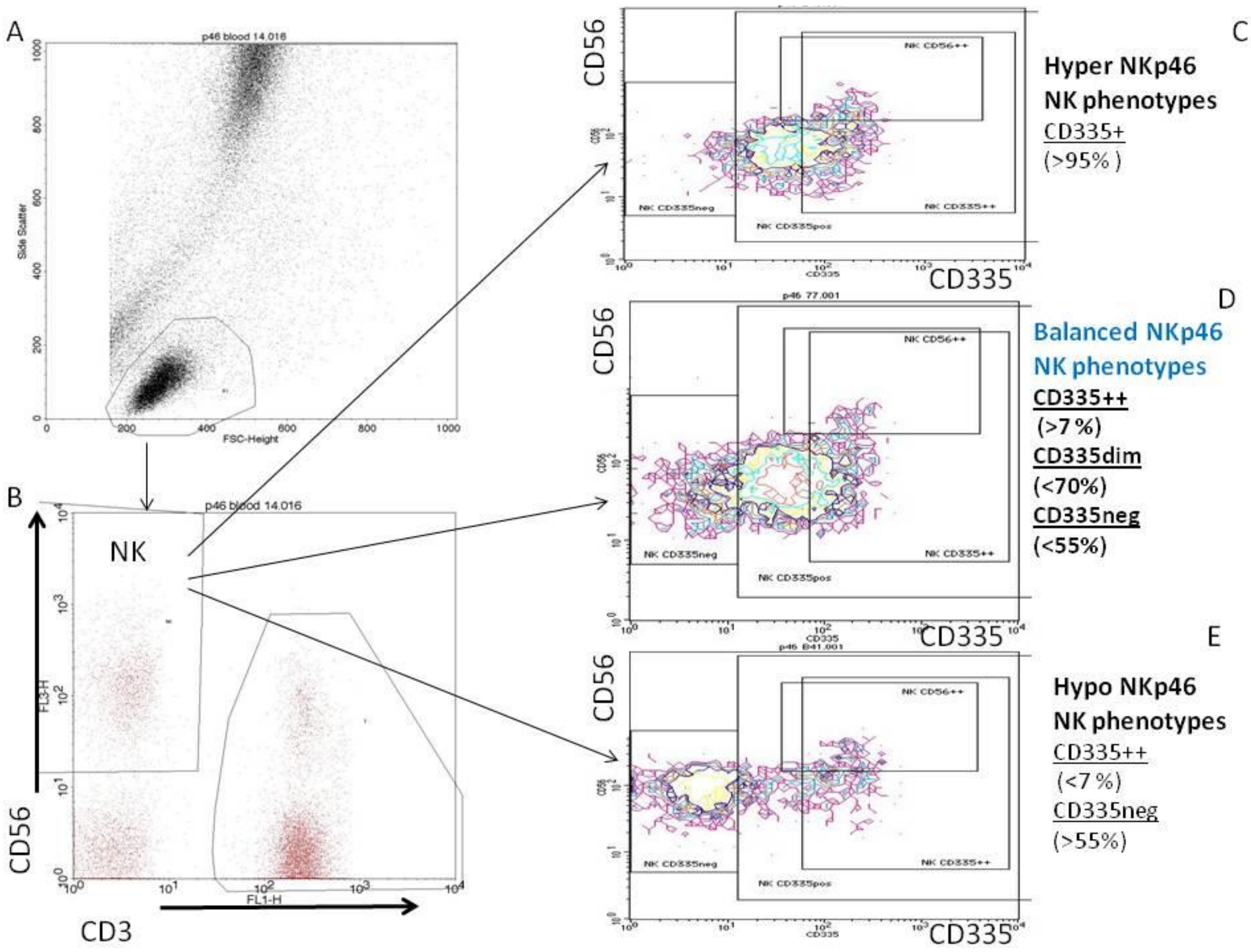

F

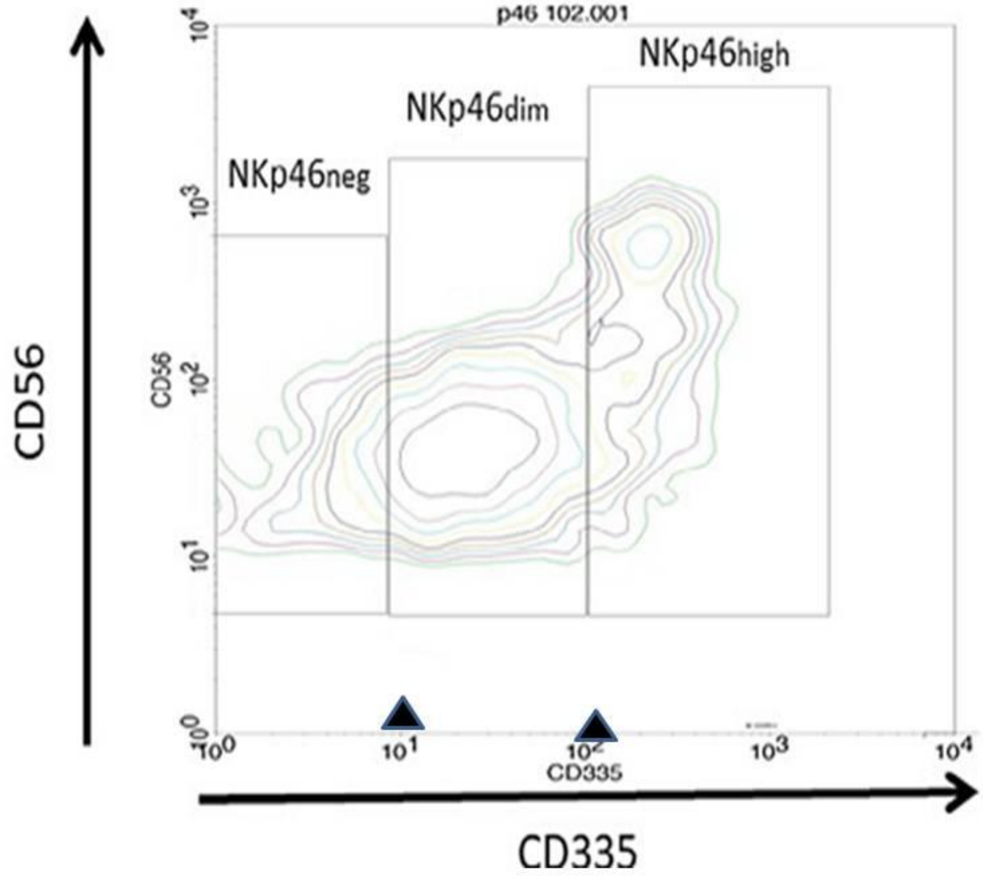

p46 102.001
NKp46high
NKp46dim
NKp46neg
CD56
CD56
CD335
CD335

Supplementary Tab. S1a

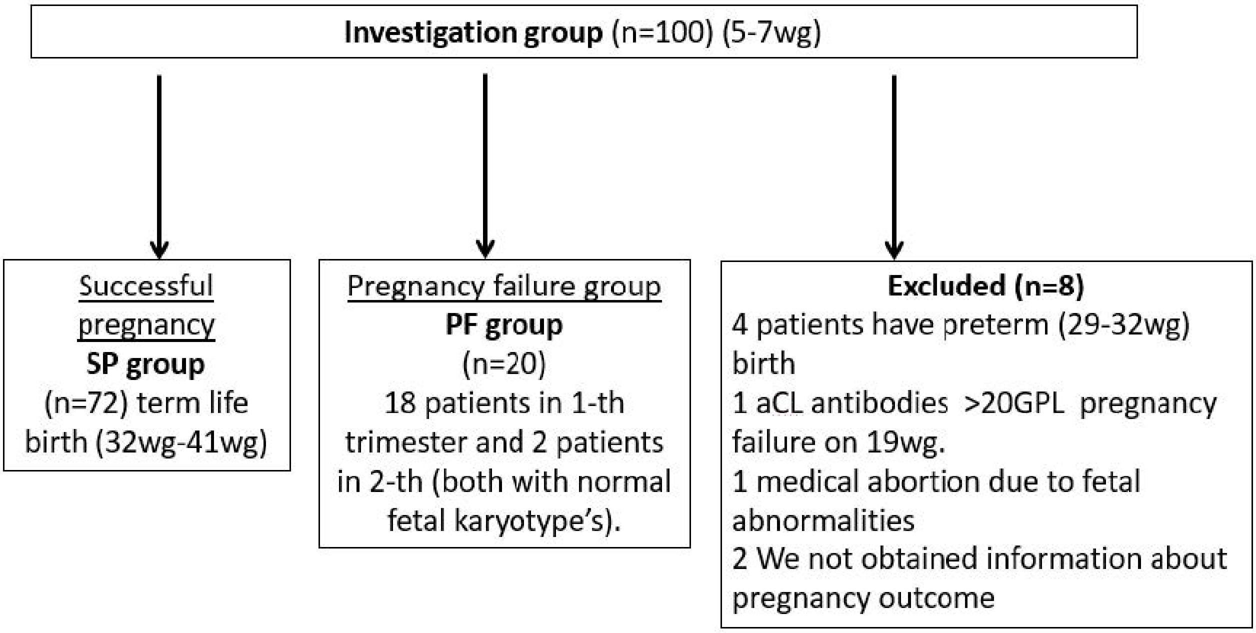

Supplementary Tab. S1b

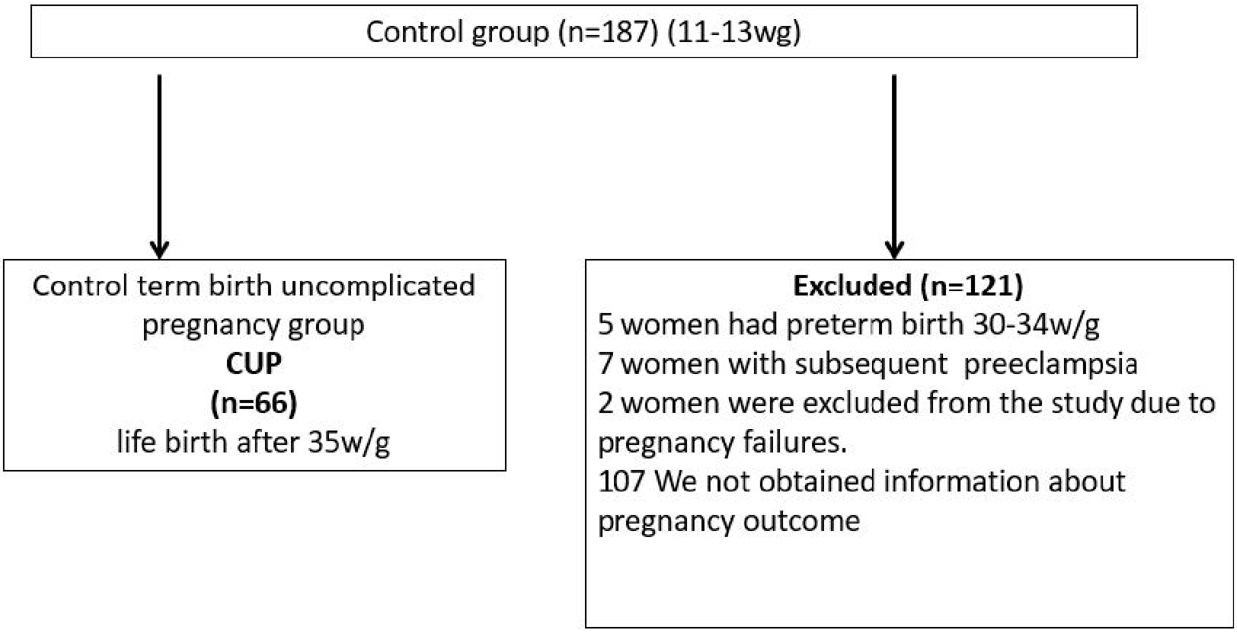

Supplement: Supplementary file 1 [file diagnostics-13-01845-s001.zip › diagnostics-2344359-supplementary.pdf]
